# Appendiceal diverticulitis: a rare pathology disguised as acute appendicitis

**DOI:** 10.1093/bjrcr/uaae047

**Published:** 2024-12-02

**Authors:** John L Chen, Venugopala Kalidindi, Jorge Mayor-Jerez, Timothy J Sadler, Daniel J Bell

**Affiliations:** Department of Radiology, North Middlesex University Hospital NHS Trust, London N18 1QX, United Kingdom; Department of Radiology, Cambridge University Hospitals NHS Foundation Trust, Cambridge CB2 0QQ, United Kingdom; School of Clinical Medicine, University of Cambridge, Cambridge CB2 0SP, United Kingdom; Department of Surgery, North Middlesex University Hospital NHS Trust, London N18 1QX, United Kingdom; Department of Histopathology, Royal Free London NHS Foundation Trust, London NW3 2QG, United Kingdom; Department of Radiology, Cambridge University Hospitals NHS Foundation Trust, Cambridge CB2 0QQ, United Kingdom; School of Clinical Medicine, University of Cambridge, Cambridge CB2 0SP, United Kingdom; Department of Radiology, North Middlesex University Hospital NHS Trust, London N18 1QX, United Kingdom

**Keywords:** appendix, vermiform appendix, acute appendicitis, appendiceal diverticulosis, appendiceal diverticulitis

## Abstract

Appendiceal diverticulitis, although rare, is an important differential diagnosis in cases of right iliac fossa pain. Previously, it has often been considered as a variant of acute appendicitis, due to its seemingly similar clinical presentation. However, recent research indicates that appendiceal diverticulitis is a distinct clinical entity, with demographic characteristics and clinical features that are different to acute appendicitis. It is also associated with higher risk of severe morbidity and mortality, necessitating timely diagnosis and management. In this report, we present a case of a 58-year-old male patient with diverticulitis of the vermiform appendix and review the relevant literature. We describe the classification of appendiceal diverticulosis and diverticulitis, their clinical presentation, and their potential complications. We outline the radiological findings of appendiceal diverticulitis and acute appendicitis and discuss the important role of diagnostic imaging in distinguishing between these 2 conditions.

## Clinical presentation

A 58-year-old man presented with a 5-day history of severe lower right abdominal pain, fever, and diarrhoea. He had no nausea or vomiting and had been able to eat and drink normally. He had a past medical history of hypertension. He had not undergone any previous abdominal procedures. He reported a history of occasional alcohol consumption and smoking. On examination, his abdomen was soft but severely tender in the right iliac fossa, with a positive Rovsing sign. His vital signs were unremarkable, including normal body temperature.

## Differential diagnosis

The most highly suspected diagnosis in cases of severe right iliac fossa pain is usually acute appendicitis. However, there also exist other forms of appendiceal pathology. These include diverticulitis of the appendix, intussusception of the appendix, appendiceal neoplasms, and incarcerated herniation of the appendix.^1^^,^^2^

In addition, a wide range of other intra-abdominal pathology can result in a similar presentation. Gastrointestinal causes include gastroenteritis, mesenteric adenitis, Meckel’s diverticulitis, intestinal intussusception, caecal diverticulosis or diverticulitis, Crohn’s disease, perforated peptic or duodenal ulceration, pancreatitis, cholecystitis or other hepatobiliary disease, right inguinal or femoral hernia, colonic carcinoma, epiploic appendagitis, intestinal obstruction, and mesenteric ischaemia. Genitourinary causes include ureteric colic, pyelonephritis, epididymo-orchitis, and testicular torsion. Gynaecological causes include ectopic pregnancy, endometriosis, haemorrhagic ovarian cyst, ovarian torsion, pelvic inflammatory disease, and fibroid degeneration.^1^

## Investigations

**Figure 1.**
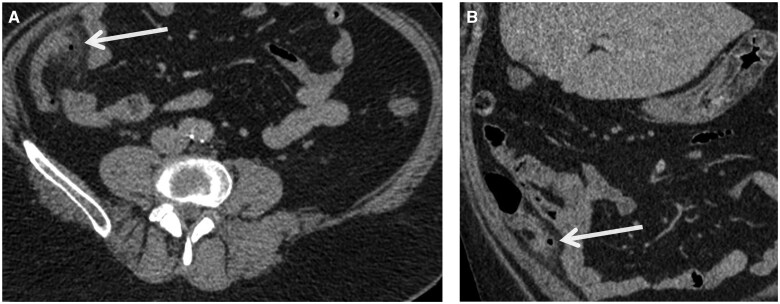
Contrast-enhanced CT of the abdomen in portal venous phase. (A) Axial section. (B) Coronal section. At the medial aspect of the appendix (arrows), there are several diverticula that are markedly inflamed and surrounded by fat stranding, consistent with appendiceal diverticulitis.

Blood tests demonstrated a normal white cell count of 9.5 × 10^9^/L (3.0-10.0 × 10^9^/L) and mildly elevated C-reactive protein (CRP) of 31 mg/L (0-5 mg/L). A contrast-enhanced CT of the abdomen and pelvis was performed in portal venous phase. This revealed several markedly inflamed diverticula at the medial aspect of the appendix, surrounded by fat stranding. The appendix itself was not distended and had a normal thin wall. There was no fluid collection. Several reactive lymph nodes were noted in the draining mesentery. Mild uncomplicated sigmoid diverticulosis was also present. Overall, these appearances were consistent with a diagnosis of appendiceal diverticulitis (Figure 1).

## Treatment

**Figure 2.**
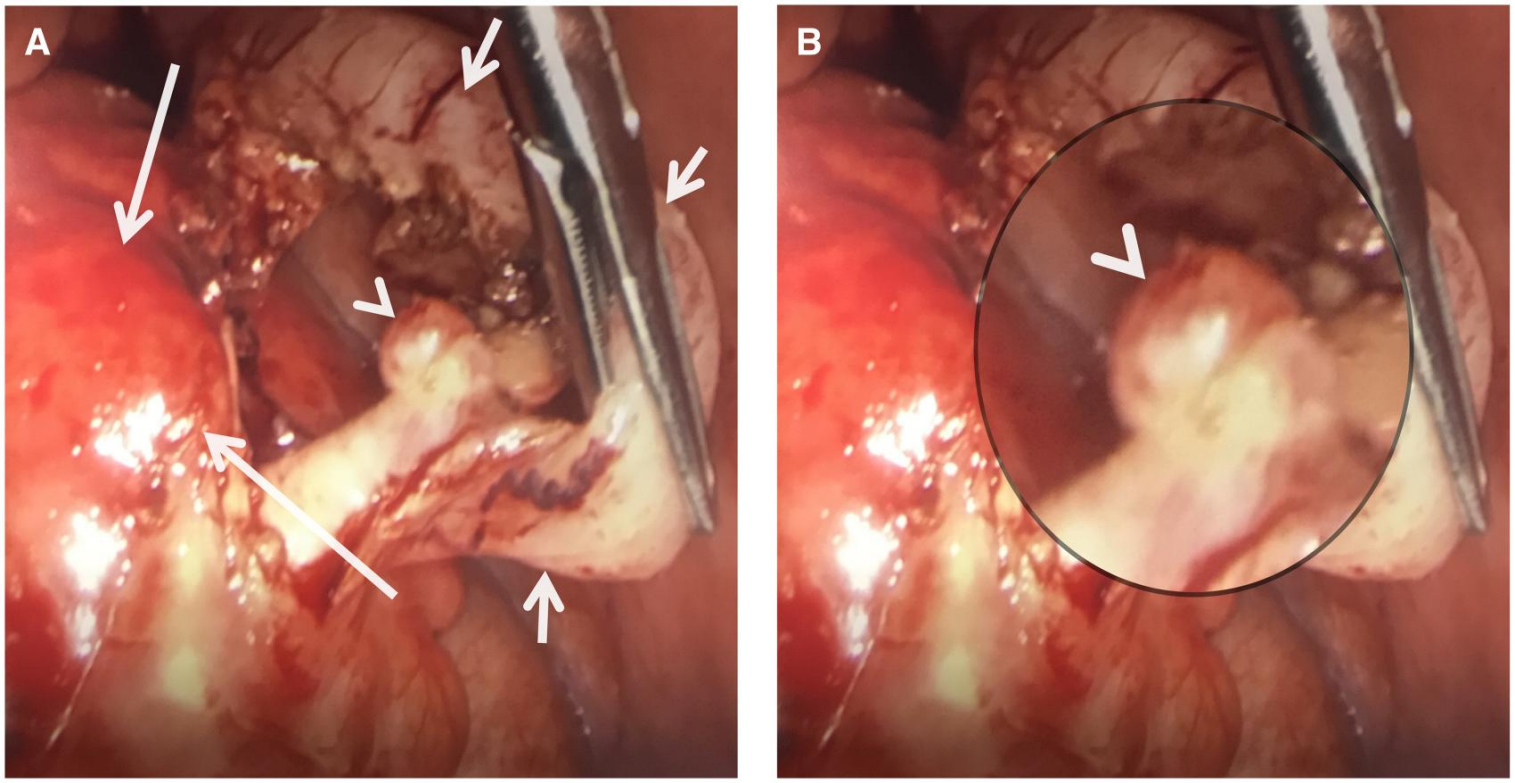
Macroscopic images from the laparoscopic appendicectomy. (A) Non-magnified view showing the caecum (long arrows) and appendix (short arrows), which has an inflamed diverticulum (arrowhead). (B) Magnified view of appendiceal diverticulum (arrowhead).

The patient was treated with emergency appendicectomy, given the degree of inflammation and propensity for perforation. The procedure was performed laparoscopically and was successful, without any complications. Operative findings confirmed the presence of multiple appendiceal diverticula, with one complicated with phlegmon. The lie of the appendix was retrocolic and the base was healthy. No other abnormalities were observed in the abdomen (Figure 2).

## Outcome

**Figure 3.**
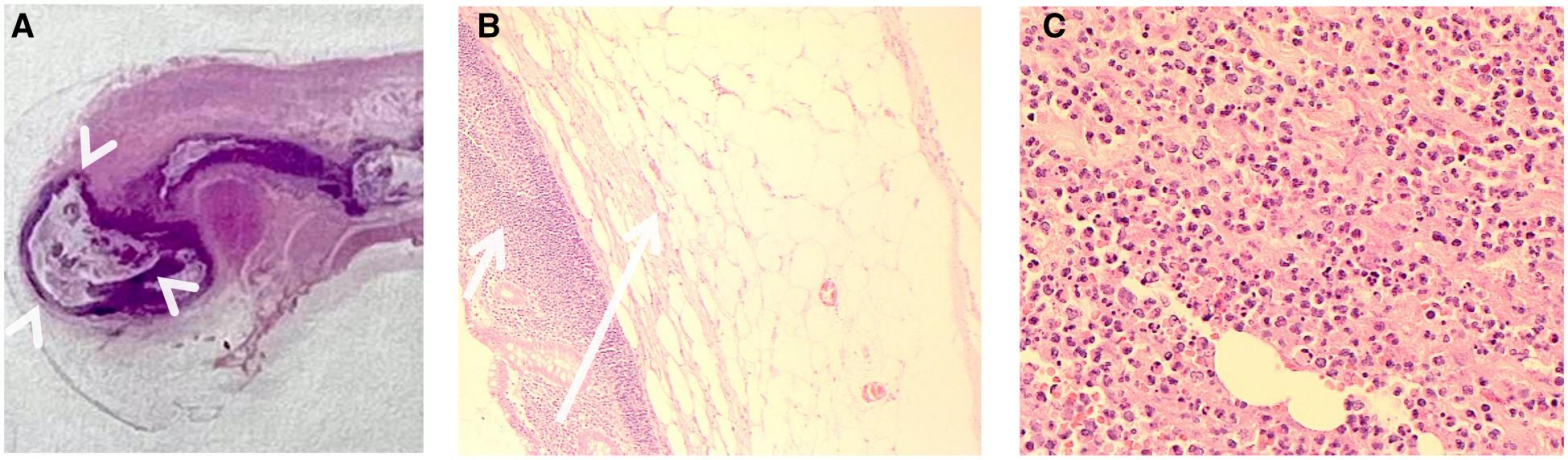
Microscopic images of the resected appendix. (A) Low-magnification image showing herniation (arrowheads) of the mucosa and muscularis mucosa through the appendix wall. (B) High-magnification image showing the herniated diverticular mucosa (short arrow) and submucosa (long arrow) without muscularis propria. (C) High-magnification image showing acute inflammation with a predominance of neutrophils, which involved all layers of the appendix wall.

Following the operation, histopathological analysis of the removed appendix demonstrated acute inflammation with diverticulitis in its tip, but no evidence of any other unusual features (Figure 3). This confirmed the diagnosis of appendiceal diverticulitis with associated appendicitis. The patient had an unremarkable post-operative course.

## Discussion

Though vestigial, the vermiform appendix is a prominent source of pathology. Besides acute appendicitis, it can be the site of numerous other inflammatory, infectious, and neoplastic conditions.

Appendiceal diverticulitis is a rare disorder in which there is acute inflammation of diverticula arising from the appendix. Diverticula are small, bulging pouches within the bowel wall.^3^ Although the appendix is itself an embryological diverticulum of the caecum, it can develop diverticula within its own wall. Diverticula of the appendix are divided into 2 histological types, congenital and acquired.^4^ Congenital, or true, diverticula consist of herniation of all layers of the appendix wall, including mucosa, submucosa, and muscularis. These are extremely rare and sometimes associated with chromosomal abnormalities. Acquired, or false, diverticula are more common, and involve herniation of only the mucosa and submucosa through a defect in the muscular layer, as in this case.^3^ Diverticulosis of the appendix is usually asymptomatic and may be discovered incidentally during operations or imaging studies, but two-thirds of patients go on to develop appendiceal diverticulitis.

Despite first being described in 1893 by a British physician, appendiceal diverticulitis is still an uncommonly recognized condition.^5^ Although it has historically been considered a rare form of pathology, a recent retrospective review of pathological specimens from appendicectomies found that the incidence was almost 10%, raising the possibility that appendiceal diverticulitis may be a far more prominent clinical problem than hitherto recognized.^3^ Appendiceal diverticulitis and diverticulosis have been classified into 4 morphological types: type 1 describes acute diverticulitis within an otherwise normal appendix; type 2 describes acute diverticulitis with surrounding appendicitis, as in this case; type 3 describes acute appendicitis with an incidental uninvolved diverticulum; and type 4 describes an incidental diverticulum with no diverticulitis or appendicitis.^6^ Types 3 and 4 involve no diverticular inflammation and therefore represent appendiceal diverticulosis rather than diverticulitis.

Although dismissed for a long time as a variant of true appendicitis, appendiceal diverticulitis has been established by recent studies as a distinct clinical entity, with different clinical features and risks.^5^ Like acute appendicitis, it usually presents with right iliac fossa pain and fever in the acutely unwell patient. However, while appendicitis often manifests with anorexia, nausea, and vomiting, these symptoms are less common in appendiceal diverticulitis, which usually presents with more intermittent pain over a longer interval.^6^ The duration of symptoms in appendiceal diverticulitis is usually around 3-4 days, compared to 1-2 days for acute appendicitis.^3^^,^^4^ Appendiceal diverticulitis also occurs in an older patient age group than acute appendicitis, with an average age of 40-50 years compared to 20-30 years for acute appendicitis.^4^ Other risk factors include male sex and a background of cystic fibrosis.^5^

There is a remarkable difference in the risk of severe complications. In appendiceal diverticulitis, perforation is 4 times more common than in acute appendicitis, resulting in a mortality rate 30 times higher.^5^ Recent retrospective reviews confirm that, among resected specimens, perforation rates are around 60%-70%, compared to around 10%-30% for acute appendicitis.^3^^,^^4^ This may be related to the vaguer presentation with an insidious onset of symptoms, meaning that patients with appendiceal diverticulitis may seek medical attention much later than those with acute appendicitis. For the same reasons, preoperative diagnosis may be more difficult, and surgical treatment may be delayed.^5^ Due to perforation by the time of operation, surgery may be more difficult, with studies showing increased operative time and intra-operative blood loss compared to acute appendicitis.^3^ Other possible complications of appendiceal diverticulitis include diverticular haemorrhage, intestinal obstruction or intussusception, formation of intraperitoneal abscesses, pelvic pseudocysts, or vesicocaecal fistulae.^4^ Appendiceal diverticulosis and diverticulitis also appear to be associated with neoplastic processes. It has been reported that almost half of appendicectomy specimens with diverticulosis harboured neoplasms.^7^ In particular, appendiceal diverticula are associated with mucinous neoplasms of the appendix, with a possible role in the pathogenesis of pseudomyxoma peritonei.^7^

These characteristics affect management considerations. The higher rate of perforation and other complications makes immediate surgical resection necessary, even if abdominal pain is not severe.^4^ The potential association with neoplastic pathology means that prophylactic appendicectomy may be advisable when diverticulosis is discovered incidentally.^7^ It is important for surgeons and pathologists to examine the affected areas meticulously, with histological inspection of the entire appendicectomy specimen to rule out any associated neoplasm.^7^

Given such a different risk profile to acute appendicitis, it is important that cases of appendiceal diverticulitis can be diagnosed in an accurate and timely manner. Recent studies suggest that these 2 conditions have distinct radiological appearances that can be used to aid their diagnostic differentiation.

Ultrasound findings of acute appendicitis consist of an anechoic fluid-filled space surrounded by echogenic oedematous mucosa, appearing as a ring in transverse section. In comparison, appendiceal diverticulitis appears with thickened and echogenic appendix wall layers due to the presence of gas within. Direct visualization of inflamed appendiceal diverticula is also possible on ultrasound.^8^

On CT, it can be more difficult to visualize the appendix clearly in appendiceal diverticulitis than in acute appendicitis.^3^ Appearances associated with appendiceal diverticulitis include peri-appendiceal extraluminal fluid, peri-appendiceal fat stranding, larger appendix calibre, formation of abscesses, absence of intraluminal fluid, and absence of appendicoliths.^3^^,^^9^ Inflamed diverticula may be visualised as small fluid-filled luminal structures with thick enhanced walls or as solid enhanced masses protruding from the appendix, while normal diverticula may be visualised as small air-filled structures with thin walls. In most cases, there is appendiceal wall thickening, but little or no intraluminal fluid collection.^10^

In conclusion, we present a case of appendiceal diverticulitis, an uncommon pathology of the appendix. Though frequently overlooked, it is an important part of the differential diagnosis of right iliac fossa pain as it is associated with significantly increased risk of perforation and appendiceal neoplasms.^6^ It is essential for clinicians and radiologists to be aware of its subtler clinical presentation and radiographic features, which are key to its accurate and timely diagnosis.

## Learning points

- Severe right iliac fossa pain has a variety of pathological causes besides acute appendicitis; likewise, the vermiform appendix can be affected by numerous pathologies other than acute appendicitis.
- Appendiceal diverticulitis is a rare diagnosis but may be more prevalent than previously recognized and has distinct demographic and clinical characteristics compared to acute appendicitis.
- Appendiceal diverticulitis is associated with higher risks of perforation and mortality and is associated with neoplasms of the appendix; management therefore involves urgent appendicectomy followed by thorough pathological examination of the resected specimen, with prophylactic appendicectomy advised in asymptomatic patients.
- Radiologists should be aware of appendiceal diverticulitis and its appearances on ultrasound and CT, to enable its early diagnostic differentiation from other forms of appendix pathology.
